# Supplementary material for: The Phenotypic Spectrum of PRRT2-Associated Paroxysmal Neurologic Disorders in Childhood
Source: Biomedicines. 2020 Oct 28;8(11):456. doi: 10.3390/biomedicines8110456 (PMC7719266; doi:10.3390/biomedicines8110456)
Supplement: Supplementary file 1 [file biomedicines-08-00456-s001.pdf]

Variant	Protein	Prediction (CADD, SIFT, PolyPhen2)	Reference	Inheritance
c.323_324del	p.(Thr108Serfs*25)	NA	Yang (2014) Zhonghua Er Ke Za Zhi 52, 806; Zeng (2018) J Hum Genet 63: 9	de novo
c.341_342del	p.(Val114Glufs*19)	NA	this study	familial
c.593_594del	p.(Pro198Argfs*26)	NA	this study	familial
c.649dupC	p.(Arg217Profs*8)	NA	Chen (2011) Nat Genet 43, 1252; Heron (2012) Am J Hum Genet 90: 152	familial
c.836C>T	p.(Pro279Leu)	25.3, deleterious (0), probably damaging (0.999)	Møller (2016) Mol Syndromol 7, 210; Zhao (2019) CNS Neurosci Ther	familial
c.843G>T	p.(Trp281Cys)	33, deleterious (0,01), probably damaging (0.997)	this study	familial
c.(?-65)_(1243-?)del	exon 2-4 del	NA	this study	de novo
16p11.2del	16p11.2 del	NA	Vlaskamp (2019) Eur J Med Genet 62, 265	de novo

All variants are absent in controls (GnomAD database)

NA: not applicable,
